# Supplementary material for: TOPAS-Tissue: A Framework for the Simulation of the Biological Response to Ionizing Radiation at the Multi-Cellular Level
Source: Int J Mol Sci. 2024 Sep 19;25(18):10061. doi: 10.3390/ijms251810061 (PMC11431975; doi:10.3390/ijms251810061)
Supplement: Supplementary file 1 [file ijms-25-10061-s001.zip › ijms-3140638-supplementary.pdf]

Supplementary Material

TOPAS-Tissue: A framework for the simulation of the biological response to ionizing radiation at the multi-cellular level.

Omar Rodrigo García García, Ramon Ortiz, Eduardo Moreno Barbosa, Naoki D-Kondo, Bruce Faddegon, Jose Ramos-Méndez

Details for the DNA repair and cellular response models

The models for the biological radiation response used in this work are based on the work of Xiao-Peng Zhang, Feng Liu, and Wei Wang (2011) "Two-phase dynamics of p53 in the DNA damage response", a refined version of a previous work [Zhang X. P., et al (2009)]. It is model that combines four different modules that handle specific aspects of the process.

The first module is the DNA repair, it is a stochastic version of the Two Lesion Kinetics (TLK) model proposed by Stewart R. D. (2001). Here, the breaks can transition between several states in a probabilistic manner. The first version, proposed by Ma L., et al (2005) on "A plausible model for the digital response of p53 to DNA"; had three states: intact DSBs (DSB), attached to a repair protein complex (DSB_C) and fixed (DSB_F) as the end state.

However, this approach doesn't take into account lethal lesion accumulation that result as an inevitable consequence of the repair process and influence on the ultimate survival of the cell. Therefore, in this work the last transition was divided into two possible outcomes: the formation of a lethal lesion (DSB_L) with probability p_{lethal} or a non-lethal lesion (DSB_N) with a probability (1- p_{lethal}). The value used for this probability was 1.5%. It comes from the fraction of γ-H2AX foci at 24h post irradiation, considered as the fraction of non-repairable DSBs. The experimentally measured value for 1 Gy gamma-ray irradiation (LET ~0.2 keV/um) was 1.35±1.25% by Asaithamby A., et al (2008).

Nevertheless, the other parameters were unchanged from the original model to maintain consistency. The model classifies the initial number of DSBs as simple, handled by a fast kinetics and denoted by the subscript (1); while complex DSBs are handled by slow kinetics and denoted by the subscript (2). The transition probabilities depend on the number of DSBs on each state, the number of available repair proteins NR, the association-dissociation rates $\mathrm{k_i}$ and the time step $\Delta\mathrm{t}$. A complete scheme for the probabilities is presented next and the values for each parameter are shown in Table S1.

$$\mathrm{P(DSB_1 \rightarrow\ DSB_{C1}) = NR[k_{fb1} + k_{cross}(DSB_1 + DSB_2)]\Delta t}$$

$$\mathrm{P(DSB_2 \rightarrow\ DSB_{C2}) = NR[k_{fb2} + k_{cross}(DSB_1 + DSB_2)]\Delta t}$$

$$\mathrm{P(DSB_{C1} \rightarrow\ DSB_1)\ =\ k_{rb1}\Delta t}$$

$$\mathrm{P(DSB_{C2} \rightarrow\ DSB_2)\ =\ k_{rb2}\Delta t}$$

$$\mathrm{P(DSB_{C1} \rightarrow DSB_{L1})\ =\ p_{lethal}k_{fix1}\ \Delta t}$$

$$\mathrm{P(DSB_{C2} \rightarrow DSB_{L2})\ =\ p_{lethal}k_{fix2}\ \Delta t}$$

$$\mathrm{P(DSB_{C1} \rightarrow DSB_{N1}) = (1 - p_{lethal})k_{fix1}\ \Delta t}$$

$$P(DSB_{C2} \rightarrow DSB_{N2}) = (1 - p_{lethal})k_{fix2}\,\Delta t$$

Table S1. parameters for the DNA repair module

Parameter	Description	value
NR	Number of repair proteins	20
k_{fb1}	Association rate for repair proteins in fast kinetics	1.3
k_{fb2}	Association rate for repair proteins in slow kinetics	0.13
k_{rb1}	Dissociation rate for repair proteins in fast kinetics	0.3
k_{rb2}	Dissociation rate for repair proteins in slow kinetics	0.03
k_{fix1}	DSB ligation rate in fast kinetics	0.02
k_{fix2}	DSB ligation rate in slow kinetics	0.002
k_{cross}	DSB binary mismatch rate	0.0007
p_{lethal}	Probability of lethal lesion induction	0.015
Δt	Time step	0.5 min

The second module is the DNA damage sensor were the number of DSBs attached to a repair protein complex (DSBc) influence the activation rate of the Ataxia Telangiectasia Mutated (ATM) protein. The total quantity of ATM is considered as constant and it exist in three forms: an inactive dimer ATM_2, an inactive monomer ATM and an active form ATM^*.

The active form of ATM activates the p53 tumor control protein, initiating a series of biochemical reactions that form the radiation response protein network. In the other hand this protein is deactivated by the action of Wip1. The equations that describe this module are presented next and the values for the parameters involved are shown on Table S2:

$$ATM_{tot} = [ATM] + 2[ATM_2] + [ATM^*]$$

$$\frac{d[ATM_2]}{dt} = 0.5 * k_{dim}\,[ATM]^2 - k_{undim}\,[ATM_2]$$

$$\frac{d[ATM^*]}{dt} = k_{acATM}\frac{DSB_C}{DSB_C + j_{nc}} \cdot \frac{[ATM]}{[ATM] + j_{acATM}}[ATM^*] - k_{deATM}\frac{[ATM^*]}{[ATM^*] + j_{deATM}}(1 + [Wip1])$$

Table S2. parameters for the DNA damage sensor module

Parameter	Description	value
ATM_{tot}	Total concentration of ATM	5.0 μM
k_{dim}	ATM dimerization rate	5.0/μM min
k_{undim}	ATM undimerization rate	1.0/ min
k_{acATM}	ATM activation rate	1.3/ min
j_{acATM}	Michaelis constant for ATM activation	1.0 μM
k_{deATM}	ATM inactivation rate	0.53/min
j_{deATM}	Michaelis constant for ATM* inactivation	2.5 μM
j_{nc}	Threshold number of DSB_C for ATM activation	5
$[ATM]_0$	Initial concentration of ATM	2.17 μM
$[ATM_2]_0$	Initial concentration of ATM_2	2.73 μM
$[ATM^*]_0$	Initial concentration of ATM*	0.10 μM

The third module is the feedback control, centered around the action of the p53 protein. It consists in several regulatory cycles, the more important consist of the p53 phosphorylation (p53*) by active ATM. The presence of active p53 promotes the production of Mdm2 that in turns degrades p53*, forming a negative feedback loop. This model considers three forms of Mdm2: nuclear (Mdm2n), unphosphorylated cytoplasmic form (Mdm2c) and phosphorylated cytoplasmic form (Mdm2cp). The equations that control this module are presented next and the parameters involve are shown in Table S3.

$$\frac{d[p53^*]}{dt} = k_{acp53_1}\frac{[ATM^*]}{[ATM^*] + j_{ATM}}[p53] - k_{dep53}[p53^*] - k_{dp53s}[Mdm2_n]\frac{[p53^*]}{j_{1p53n} + [p53^*]}$$

$$\frac{d[p53]}{dt} = k_{Sp53} - k_{dp53n}[p53] - k_{dp53}[Mdm2_n]\frac{[p53]}{j_{1p53n} + [p53]} - k_{acp53_1}\frac{[ATM^*]}{[ATM^*] + j_{ATM}}[p53] + k_{dep53}[p53^*]$$

$$\frac{d[Mdm2_c]}{dt} = k_{sMdm2_0} + k_{sMdm2}\frac{[p53^*]^4}{j_{p53}^4 + [p53^*]^4} + k_{1Mdm2_s}\frac{[Mdm2_{cp}]}{j_{1Mdm2_s} + [Mdm2_{cp}]} - k_{dMdm2}[Mdm2_c] - k_{Mdm2_s}[Mdm2_c]\frac{[Aktp]}{j_{Mdm2_s} + [Mdm2_c]}$$

$$\frac{d[Mdm2_{cp}]}{dt} = k_{Mdm2_s}[Mdm2_c]\frac{[Akt_p]}{j_{Mdm2_s} + [Mdm2_c]} - k_{1Mdm2_s}\frac{[Mdm2_{cp}]}{j_{1Mdm2_s} + [Mdm2_{cp}]} - k_i[Mdm2_{cp}] + k_o[Mdm2_n] - k_{dMdm2}[Mdm2_{cp}]$$

$$\frac{d[Mdm2_n]}{dt} = k_i[Mdm2_{cp}] - k_o[Mdm2_n] - \left(k_{dMdm2_{n0}} + k_{dMdm2_{n1}}\frac{[ATM^*]}{[ATM^*] + j_{ATM}}\right)[Mdm2_n]$$

$$\frac{d[Akt^*]}{dt} = k_{acAkt}[PIP_3]\frac{[Akt]}{j_{acAkt} + [Akt]} - k_{deAkt}[PIP_3]\frac{[Akt^*]}{j_{deAkt} + [Akt^*]}$$

$$[Akt_{tot}] = [Akt] + [Akt^*]$$

$$\frac{d[PIP3]}{dt} = k_{p2}\frac{[PIP_2]}{[PIP_2] + j_{p2}} - k_{p3}[PTEN]\frac{[PIP_3]}{[PIP_3] + j_{p3}}$$

$$[PIP_2] = [PIP_{tot}] - [PIP_3]$$

Table S3. parameters for the feedback control module

Parameter	Description	Value
$k_{dMdm2_{n0}}$	Basal degradation rate of Mdm2	0.003/min
$k_{dMdm2_{n1}}$	ATM-dependent degradation rate of Mdm2	0.05/min
j_{ATM}	Michaelis constant of ATM* as a kinase	1.0μM
k_{acp53_1}	ATM-dependent activation rate of p53	0.2/ min
k_{dep53}	Deactivation rate of p53	0.1/ min
k_{dp53_s}	Mdm2-dependent degradation rate of p53*	0.01/ min
k_{sp53}	Production rate of p53	0.2μM/ min
k_{dp53_n}	Basal degradation rate of p53	0.05/ min
k_{dp53}	Mdm2-dependent degradation rate of p53	0.7/ min
j_{1p53_n}	Michaelis constant of Mdm2-dependent p53 degradation	0.1μM
k_{sMdm2_0}	Basal production rate of Mdm2	0.002μM/min
k_{sMdm2}	p53-dependent production rate of Mdm2	0.024μM/min
j_{sMdm2}	Michaelis constant of p53-induced Mdm2 production	1.0μM
k_{dMdm2_c}	Mdm2*c* degradation rate	0.003/min
k_{1Mdm2_s}	Dephosphorylation rate of cytoplasmic Mdm2	0.3μM/min
j_{1Mdm2_s}	Michaelis constant of Mdm2 dephosphorylation	0.1μM
k_{Mdm2_s}	Akt-dependent phosphorylation rate of cytoplasmic Mdm2	8/min
j_{Mdm2_s}	Michaelis constant of Akt-dependent Mdm2 dephosphorylation	0.3μM
k_i	Nuclear import rate of Mdm2cp	0.06/min
k_o	Nuclear export rate of Mdm2n	0.09/min
k_{acAkt}	*Akt* phosphorylation rate	0.25/min
j_{acAkt}	Michaelis constant for Akt phosphorylation	0.1 μM
k_{deAkt}	Akt* dephosphorylation rate	0.1μM/min
j_{deAkt}	Michaelis constant for Akt* dephosphorylation	0.2 μM
k_{p2}	PIP2 phosphorylation rate	0.1μM/min
j_{p2}	Michaelis constant for PIP2 phosphorylation	0.2μM
k_{p3}	PTEN dependent PIP3 dephosphorylation rate	0.5/min
j_{p3}	Michaelis constant for PIP3 dephosphorylation	0.4μM
PIP_{tot}	Total concentration of PIP2 and PIP3	1.0μM
Akt_{tot}	total concentration of all forms of Akt	1.0μM
$[p53]_0$	Initial concentration of inactive p53	0.8 μM
$[p53^*]_0$	Initial concentration of active p53	0.0001
$[Mdm2_c]_0$	Initial concentration of cytoplasmic Mdm2	0.1
$[Mdm2_{cp}]_0$	Initial concentration of phosphorylated cytoplasmic Mdm2	0.4
$[Mdm2_n]_0$	Initial concentration of nuclear Mdm2	0.26
$[Akt]_0$	Initial concentration of inactive Akt	0.06
$[Akt^*]_0$	Initial concentration of active Akt	0.94
$[PIP_2]_0$	Initial concentration of PIP_2	0.11 μM
$[PIP_3]_0$	Initial concentration of PIP_3	0.89 μM
$[PTEN]_0$	Initial concentration of PTEN	0.1 μM

The last module is the cell fate decision. The model considers two levels of p53 phosphorylation, the primary form is $p53_{arrester}$ which induces cell cycle arrest and the production of p21 and Wip1, this last one deactivates ATM* and regulates the activation of

p53 consequently. The second level of p53 phosphorylation is $p53_{killer}$ which activates p53DINP1, PTEN and p53AIP1, the last one continues the apoptotic pathway until Casp3 is activate, which is considered as the apoptosis condition in this model. The equations that describe this module are shown below and their parameters are presented on Table S4.

$$\frac{d[p53_{killer}]}{dt} = k_{p46}[p53DINP1]\frac{[p53_{arrester}]}{j_{p46} + [p53_{arrester}]} - k_{dp46}[Wip1]\frac{[p53_{killer}]}{j_{dp46} + [p53_{killer}]}$$

$$[p53_{arrester}] = [p53^{*}] - [p53_{killer}]$$

$$\frac{d[Wip1]}{dt} = k_{sWip1_0} + k_{sWip1}\frac{[p53_{arrester}]^3}{j_{sWip1}^3 + [p53_{arrester}]^3} - k_{dWip1}[Wip1]$$

$$\frac{d[p53DINP1]}{dt} = k_{sDINP1_0} + k_{sDINP1_1}\frac{[p53_{arrester}]^3}{j_{sDINP1_1}^3 + [p53_{arrester}]^3} + k_{sDINP1_2}\frac{[p53_{killer}]^3}{j_{sDINP1_2}^3 + [p53_{killer}]^3} - k_{dDINP1}[p53DINP1]$$

$$\frac{d[PTEN]}{dt} = k_{sPTEN_0} + k_{sPTEN}\frac{[p53_{killer}]^3}{[p53_{killer}]^3 + j_{PTEN}^3} - k_{dPTEN}[PTEN]$$

$$\frac{d[p21]}{dt} = k_{sp21_1} + k_{sp21}\frac{[p53_{arrester}]^3}{j_{sp21}^3 + [p53_{arrester}]^3} - k_{dp21}[p21]$$

$$\frac{d[p53AIP1]}{dt} = k_{sAIP1} + k_{sAIP1}\frac{[p53_{killer}]^3}{j_{sAIP1}^3 + [p53_{killer}]^3} - k_{dAIP1}[p53AIP1]$$

$$\frac{d[CytoC]}{dt} = \left(k_{acCytoC_1} + k_{acCytoC_2}[p53AIP1]\frac{[Casp3]^4}{[Casp3]^4 + j_{Casp3}^4}\right)(CytoC_{tot} - [CytoC]) - k_{deCytoC}[CytoC]$$

$$\frac{d[Casp3]}{dt} = \left(k_{acCasp3_0} + k_{acCasp3_1}\frac{[CytoC]^4}{[CytoC]^4 + j_{cytoC}^4}\right)([Casp3_{tot}] - [Casp3]) - k_{deCasp3}[Casp3]$$

Table S4. parameters for the cell fate decision module

Parameter	Description	Value
k_{p46}	phosphorylation rate of p53 arrester	$0.6/min$
j_{p46}	Michaelis constant of p53 arrester phosphorylation	$0.5\mu M$
k_{dp46}	dephosphorylation rate of p53 killer	$0.3/min$
j_{dp46}	Michaelis constant of p53 killer dephosphorylation	$0.2\mu M$
k_{sWip1_0}	Basal induction rate of Wip1	$0.01\mu M/min$
k_{sWip1}	production rate of Wip1 induced by p53 arrester	$0.09\mu M/min$

j_{sWip1}	Michaelis constant of p53 dependent Wip1 production	$0.5\mu M$
k_{dWip1}	Degradation rate of Wip1	$0.05/min$
k_{sDINP1_0}	Basal induction rate of p53DINP1	$0.001\mu M/min$
k_{sDINP1_1}	production rate of p53DINP1 induced by p53 arrester	$0.01\mu M/min$
j_{sDINP1_1}	Michaelis constant for p53 arrester dependent p53DINP1 production	$0.7\mu M$
k_{sDINP1_2}	production rate of p53DINP1 induced by p53 killer and E2F1	$0.07\mu M/min$
j_{sDINP1_2}	Michaelis constant for p53 killer dependent p53DINP1 production	$0.3\mu M$
k_{dDINP1}	Degradation rate of p53DINP1	$0.01/min$
k_{sPTEN_0}	Basal induction rate of PTEN	$0.01\mu M/min$
k_{sPTEN}	p53 killer inducible production rate of PTEN	$0.5\mu M/min$
j_{sPTEN}	Michaelis constant of p53 dependent PTEN production	$1.0\mu M$
k_{dPTEN}	Degradation rate of PTEN	$0.1/min$
k_{sp21_0}	Basal induction rate of p21	$0.01\mu M/min$
k_{sp21}	production rate of p21 induced by p53 arrester	$0.2\mu M/min$
j_{sp21}	Michaelis constant of p53 arrester inducible p21 production	$0.6\mu M$
k_{dp21}	Degradation rate of p21	$0.1/ min$
k_{sAIP1_0}	Basal induction rate of p53AIP1	$0.01\mu M/min$
k_{sAIP1}	production rate of p53AIP1 induced by p53 killer	$0.32\ \mu M/min$
j_{sAIP1}	Michaelis constant of p53 dependent p53AIP1 production	$1.5\mu M$
k_{dAIP1}	Degradation rate of p53AIP1	$0.1/min$
$k_{acCytoC_0}$	Basal release rate of mitochondrial cytochrome C	$0.001\ /min$
$k_{acCytoC_1}$	p53AIP1 dependent release rate of mitochondrial cytochrome C	$0.9\ /min$
j_{Casp3}	Michaelis constant of caspase 3-dependent cytochrome C release	$0.5\mu M$
$k_{deCytoC}$	Mitochondrial influx rate of cytochrome C	$0.05/min$
$CytoC_{tot}$	Total cytochrome C concentration	$3.0\mu M$
$Casp3_{tot}$	Total caspase 3 concentration	$3.0\ \mu M$
$k_{acCasp3_0}$	Basal activation rate of caspase 3	$0.001\mu M/min$
$k_{acCasp3_1}$	Activation rate of caspase 3	$0.9\mu M/min$
j_{CytoC}	Michaelis constant of cytochrome C dependent caspase 3 activation	$0.5\mu M$
$k_{deCasp3}$	Inactivation rate of caspase 3	$0.07\ \mu M/min$
$[p53_{arrester}]_0$	Concentration of primarily phosphorylated p53 (arrester)	$0.0\ \mu M$
$[p53_{killer}]_0$	Concentration of further phosphorylated p53 (killer)	$0.0\ \mu M$
$[Wip1]_0$	Concentration of wild-type p53 induced protein 1 (Wip1)	$0.2\ \mu M$
$[p53DINP1]_0$	Concentration of p53-dependent damage-inducible protein 1	$0.0\ \mu M$
$[p53AIP1]_0$	Concentration of p53-regulated Apoptosis-Inducing protein 1	$0.1\ \mu M$
$[CytoC]_0$	concentration of cytochrome C	$0.06\ \mu M$
$[Casp3]_0$	Concentration of active caspase 3	$0.05\ \mu M$

A scheme of how all the systems on the simulations interact is presented next on figure S1.

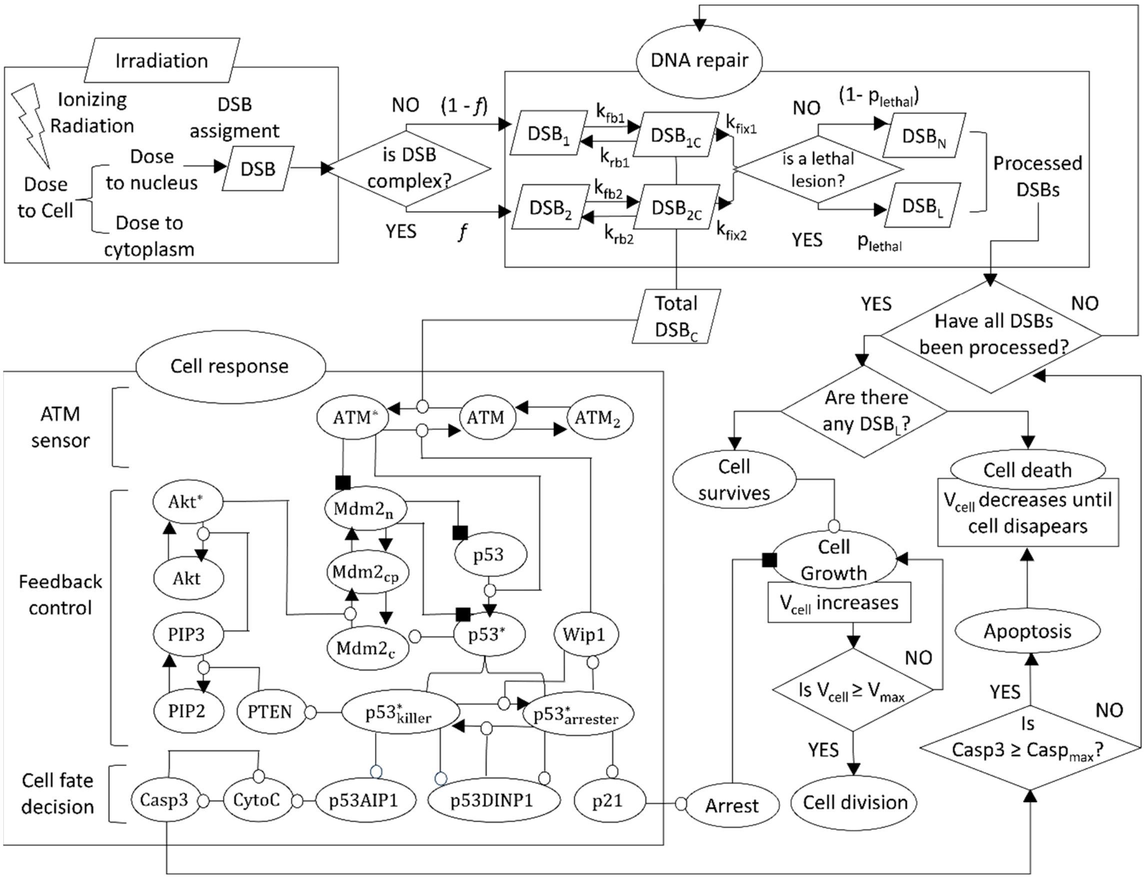

Figure S1. Complete scheme of all the mechanisms working together in TOPAS-Tissue, the cell response diagram was adapted from Zheng X.P. et al., (2011). White ellipses represent biological process and molecules, white trapezoids represent irradiation process and products, and diamonds represent decisions. Solid lines with arrowheads represent a state transition, solid lines with white circles represent a promotion of a process or biomolecule while black squares represent an inhibition of a process or degradation of a biomolecule. Finally, double arrows represent the end state of a process.

We performed test to ensure that the TOPAS-Tissue implementation of the p53 reaction network model was correct and compare the pulse profile of the active ATM, p53* and Wip1 from our simulations with the one reported on Zheng X. P. et al (2011).

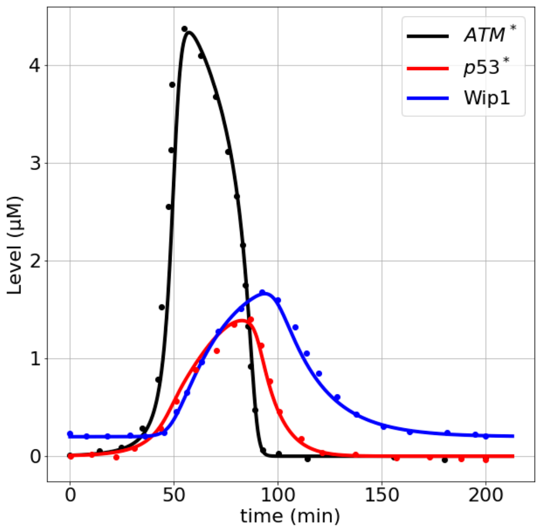

Figure S2: comparison between ATM*, p53* and Wip1 pulses from TOPAS-Tissue implementation (solid lines) and published data from Zheng X. P., et al (2011) (dots)

We also ensure that the interval between p53 pulses (approximately 360 min or 6hr) and the amplitude (around 4.6μM) corresponded with the values reported in the original publication.

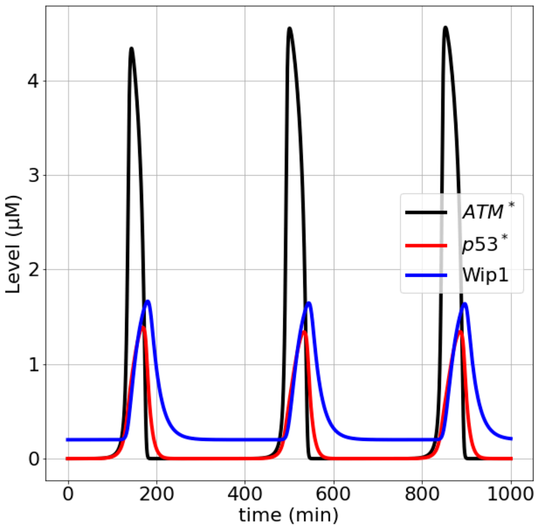

Figure S3: train of ATM*, p53* and Wip1 pulses.

The Monte Carlo repair model was also tested against a TLK equivalent model solved numerically with a 4th degree Runge-Kutta integrator: we perform 10 repetitions with 122 initial DSBs and a complex DSB fraction of 0.51.

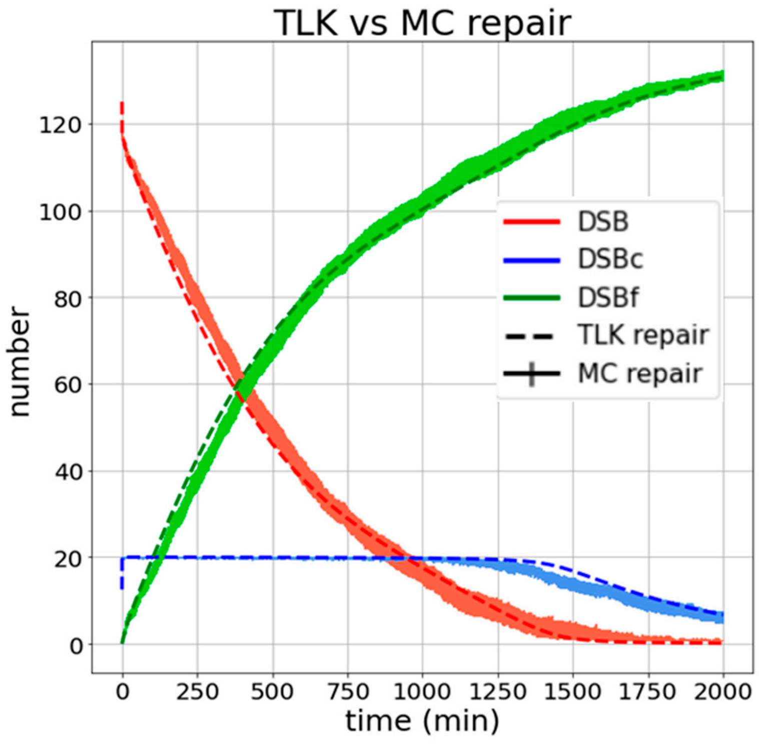

Figure S4: comparison between TLK (dash line) with the MC repair mechanism.

Bibliography

Stewart, R.D. Two-lesion kinetic model of double-strand break rejoining and cell killing. Radiat. Res. 2001, 156, 365–378. https://doi.org/10.1667/0033-7587(2001)156[0365:tlkmod]2.0.co;2.

Ma, L.; Wagner, J.; Rice, J.J.; Hu, W.; Levine, A.J.; Stolovitzky, G.A. A plausible model for the digital response of p53 to DNA damage. Proc. Natl. Acad. Sci. USA 2005, 102, 14266–14271. https://doi.org/10.1073/pnas.0501352102.

Zhang, X.-P.; Liu, F.; Cheng, Z.; Wang, W. Cell fate decision mediated by p53 pulses. Proc. Natl. Acad. Sci. USA 2009, 106, 12245–12250. https://doi.org/10.1073/pnas.0813088106.

Zhang, X.-P.; Liu, F.; Wang, W. Two-phase dynamics of p53 in the DNA damage response. *Proc. Natl. Acad. Sci. USA* **2011**, *108*, 8990–8995. https://doi.org/10.1073/pnas.1100600108

Asaithamby, A.; Uematsu, N.; Chatterjee, A.; Story, M.D.; Burma, S.; Chen, D.J. Repair of HZE-Particle-Induced DNA Dou-ble-Strand Breaks in Normal Human Fibroblasts. Radiat. Res. 2008, 169, 437–446. https://doi.org/10.1667/rr1165.1.
